# A cluster randomized-controlled trial of a community mobilization intervention to change gender norms and reduce HIV risk in rural South Africa: study design and intervention

**DOI:** 10.1186/s12889-015-2048-z

**Published:** 2015-08-06

**Authors:** Audrey Pettifor, Sheri A. Lippman, Amanda M Selin, Dean Peacock, Ann Gottert, Suzanne Maman, Dumisani Rebombo, Chirayath M. Suchindran, Rhian Twine, Kathryn Lancaster, Tamu Daniel, F. Xavier Gómez-Olivé, Kathleen Kahn, Catherine MacPhail

**Affiliations:** Department of Epidemiology, University of North Carolina Gillings School of Global Public Health, Chapel Hill, NC USA; Honorary appointments at MRC/Wits Rural Public Health and Health Transitions Research Unit (Agincourt) and Wits Reproductive Health and HIV Institute (WHRI), University of the Witwatersrand, Johannesburg, South Africa; Center for AIDS Prevention Studies (CAPS), Department of Medicine, University of California at San Francisco, San Francisco, CA USA; Carolina Population Center, University of North Carolina at Chapel Hill, Chapel Hill, NC USA; Sonke Gender Justice, Cape Town, South Africa; Department of Health Behavior, University of North Carolina Gillings School of Global Public Health, Chapel Hill, NC USA; Department of Biostatistics, University of North Carolina Gillings School of Global Public Health, Chapel Hill, NC USA; MRC/Wits Rural Public Health and Health Transitions Research Unit (Agincourt), School of Public Health, Faculty of Health Sciences, University of the Witwatersrand, Johannesburg, South Africa; US Department of State, Lusaka, Zambia; CRN for Mental Health and Wellbeing, University of New England, Armidale, NSW Australia

## Abstract

**Background:**

Community mobilization (CM) interventions show promise in changing gender norms and preventing HIV, but few have been based on a defined mobilization model or rigorously evaluated. The purpose of this paper is to describe the intervention design and implementation and present baseline findings of a Cluster Randomized Controlled Trial (RCT) of a two-year, theory-based CM intervention that aimed to change gender norms and reduce HIV risk in rural Mpumalanga province, South Africa.

**Methods:**

Community Mobilizers and volunteer Community Action Teams (CATs) implemented two-day workshops, a range of outreach activities, and leadership engagement meetings. All activities were mapped onto six theorized mobilization domains. The intervention is being evaluated by a randomized design in 22 communities (11 receive intervention). Cross-sectional, population-based surveys were conducted with approximately 1,200 adults ages 18–35 years at baseline and endline about two years later.

**Conclusions:**

This is among the first community RCTs to evaluate a gender transformative intervention to change norms and HIV risk using a theory-based, defined mobilization model, which should increase the potential for impact on desired outcomes and be useful for future scale-up if proven effective.

**Trial registration:**

ClinicalTrials.gov NCT02129530

## Background

Social environments and social norms that are not supportive of safer sexual behaviors place young men and women at extremely high risk of HIV/STI acquisition [1–3]. In South Africa social constructions of masculinity and femininity have been demonstrated to increase risk of HIV infection and other negative health outcomes, such as intimate partner violence and alcohol abuse [4–9]. Norms of masculinity in this setting place value on multiple sexual partnerships and condone physical and sexual violence as a means of establishing power in relationships with female partners [6, 7, 9]. Further, norms of masculinity often deter men from accessing health services, resulting in poor uptake of preventive care services, including HIV testing and care [10–15].

Gender transformative interventions, which seek to reconfigure gender roles in the direction of more gender equitable relationships, [16] have demonstrated some success in changing norms and reducing risk behaviors [17–19]. While numerous gender transformative interventions have been undertaken worldwide, only a small number have been rigorously evaluated using an experimental design [17, 18]. Evaluations suggest that combining group education with Community Mobilization (CM) strategies, or those that engage communities to dialogue and take action around shared concerns, [20] may be more effective in changing norms and risk behaviors than group education alone [17, 18, 21]. CM strategies have also shown promise in creating more supportive social environments and social norms to reduce HIV risk and promote safer sexual behaviors [3, 17, 22–30]. To date, however, very few gender transformative programs with a CM approach have been designed around a defined theoretical model of community mobilization.

The aim of this paper is to describe the intervention and study design of a Cluster Randomized Controlled Trial (RCT) of a novel, theory-based CM intervention to change gender norms that place communities at risk of HIV acquisition. The two-year trial was implemented from May 2012 to June 2014 in the rural Bushbuckridge sub-district in Mpumalanga province of South Africa. This is among the first community RCTs to evaluate a gender transformative intervention to change negative gender norms and HIV risk, and to our knowledge, is the first to do so using a defined mobilization model [20, 31].

## Methods

### Intervention design

The primary objectives of the CM intervention were to increase awareness about the relationship between gender inequities and HIV and encourage the community and especially men to take action to address negative gender norms and HIV risk. The CM intervention was designed in partnership with the South African non-governmental organization Sonke Gender Justice, based on their One Man Can (OMC) Campaign. OMC was originally designed in 2006 as a program to promote healthy, equitable relationships and support men and boys to take action to end domestic and sexual violence. Trained community mobilizers carry out intensive two-day workshops on themes related to gender and health; a range of community outreach activities; and assist in establishing and training volunteer cadres called Community Action Teams (CATs) in each community (www.genderjustice.org.za). These intervention elements then become a catalyst to mobilize community members to take action in their own homes and neighborhoods.

**Fig. 1:**
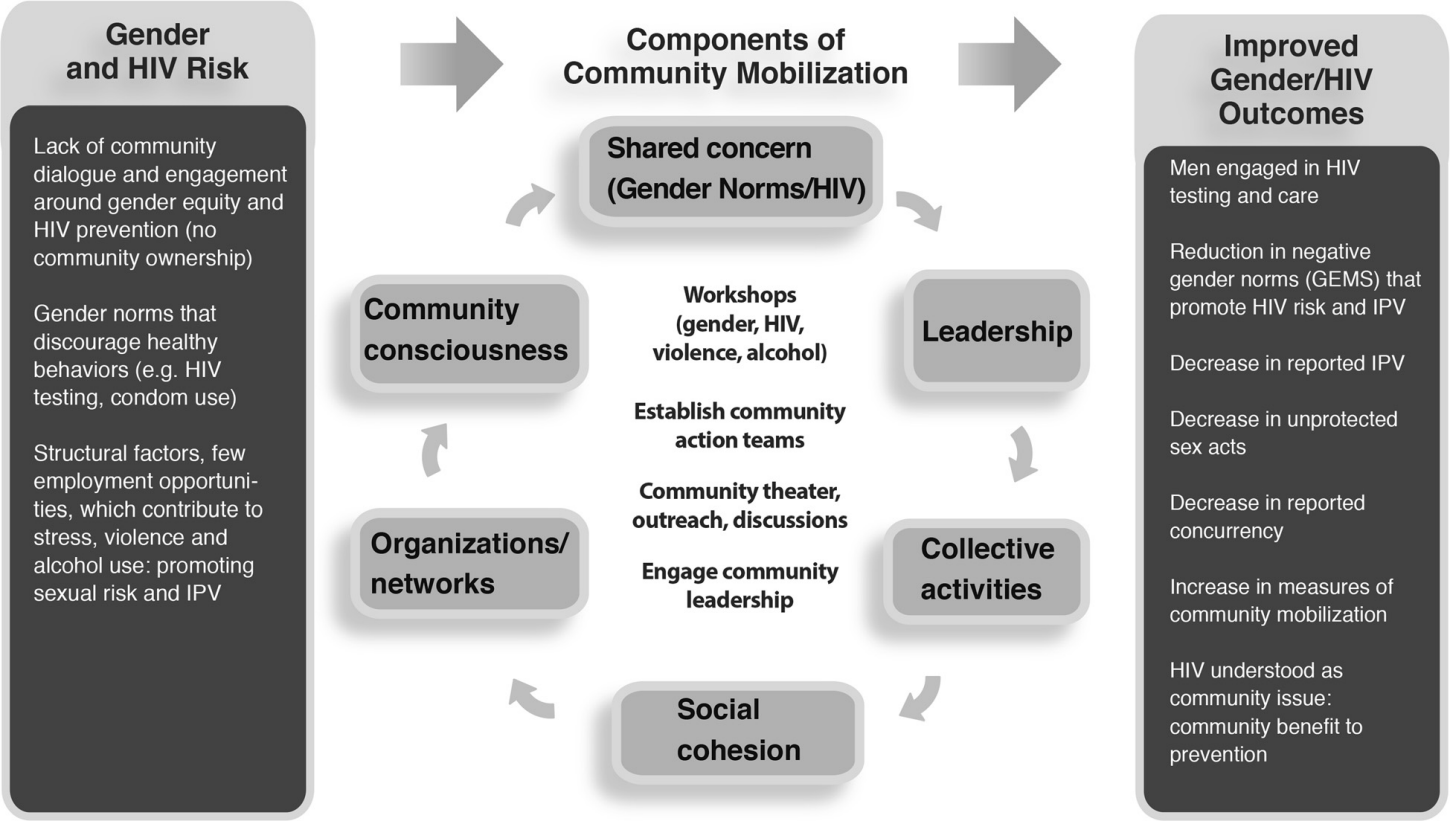
Conceptual framework of the intervention, community mobilization domains, and outcomes

The CM intervention included similar workshop structure and types of community activities, implementation by mobilizers and emphasis on CAT engagement, however for this study, thematic content and activities were extended more specifically to address HIV risk and a more a direct focus on engaging community leadership was added. Aspects of the OMC campaign that included use of the mass media for advocacy work were not included in the CM intervention, in efforts to avoid intervention contamination in comparison communities. All intervention components were carefully mapped onto a theoretical framework for CM developed through extensive formative work by the investigative team, comprising six targeted domains. These CM domains include: 1) development of a shared concern (around HIV and gender norms); 2) building critical consciousness; 3) establishing and leveraging organizations and groups, including links to networks; 4) engaging leadership (individual and/or institutional); 5) engaging communities in collective activities/actions; and 6) building social cohesion [20]. The theoretical framework is included in Fig. 1.

The Intervention was implemented by a team of two supervisors, 15 community mobilizers, and at least one CAT in each community, which on average included about eight members. Community mobilizers were selected from among men and women ages 18–35 in the communities receiving the CM intervention. Mobilizers underwent an intensive initial month-long training as well as ongoing training led by the CM intervention manager (DR) who had over 10 years of experience conducting gender awareness and mobilization-related community work. Mobilizers in turn were responsible for conducting initial and refresher trainings for CAT members, with guidance from the CM intervention managers.

Intervention workshops included both men and women and addressed seven content areas, including (1) gender, power, and health, (2) gender and violence, (3) alcohol, (4) gender, HIV and AIDS, (5) healthy relationships, (6) human rights, and (7) taking action for change. Mobilizers and CATs also created opportunities for community dialogue about these themes outside of workshops through community activities focusing on developing a shared concern and critical consciousness around HIV and gender. Examples of community activities included door-to-door home visits, street soccer and soccer tournaments, mural design and discussions, “digital story” (short testimonial film) and other film screenings with a thematic discussion to follow, health talks, and ambush theater (in which actors act out a scene in a public place, then reveal themselves and engage the crowd in conversation over the scene they witnessed). The team also engaged formal leadership in each community to discuss the intervention themes and seek support and collaboration for activities applicable to the local context of each community. A matrix showing how each of these intervention components maps onto the theorized CM domains is included in Table 1.

**Table 1:** Intervention activities mapped onto Community Mobilization domains

	Shared concerns	Community conscious-ness	Orgs/ Networks	Leadership	Collective Action	Social cohesion
Establishing Community Action Teams (CATs)			X	X		X
2-day small group workshops	X	X				X
Engaging leadership			X	X		
Community activities						
• Mini (2–3 hour) small group workshops	X	X		X (leaders workshop)		X
• Shebeen (tavern) workshops	X	X	X			
• Ambush theater	X	X				
• Door to door outreach	X	X				
• Digital stories screenings	X	X				
• Community murals					X	X
• Street soccer	X	X				
• Soccer tournaments			X	X	X	X
• Debates	X	X				
• Community events/ forums/ cultural event	X	X	X		X	X
• Film screenings with discussion	X	X				
• PhotoVoice workshops	X	X		X	X	X

All CM intervention activities were manualized and implemented with systematic targets for implementation in terms of intervention dose delivered and intervention reach within the target population. The target audience for the intervention was men and women ages 18–35 (the resident target population was approximately 25,000), although all adult community members were welcomed in workshops and community activities and events.

### Monitoring intervention implementation

Monitoring data was used to track intervention dose delivered, reach and activity mix. Data was captured using paper-based monitoring forms completed by the community mobilizers and CATS and entered into a database weekly. Targets for dose delivered for each community differed slightly by community size (larger vs. smaller communities) and included 1–2 workshops per month, 4–8 activities per week (16–32 activities per month), and 2 leadership engagement meetings per month (reduced to 1 in year 2). Targets were also set to reach 20 % of the male population age 18–35 living in the intervention communities (overall and by community) with at least one workshop in the first year and 40 % by the end of the second year. Reaching 40 % of the population by the end of the intervention was chosen as a potentially appropriate target as it is nearly three times the 15 % commonly thought of as a ‘critical mass’ of a population’s opinion leaders for successful diffusion of health behavior change messages [32]. Activity mix was monitored to ensure all content themes and mobilization domains were addressed.

### Study setting

The study setting for this community RCT includes 22 villages in the Agincourt Health and Socio-Demographic Surveillance Site (Agincourt HDSS) in the rural Bushbuckridge sub-district in Mpumalanga province of South Africa [33]. The study site is located about 500 km northeast of Johannesburg, near South Africa’s border with Mozambique. HIV prevalence in Mpumalanga is estimated to be 21.8 % among adults ages 15-49 [34] and prevalence among 35–39 year olds in the study area was over 45 % in 2010–2011 [35].

### Study design

This study employed a community randomized design to evaluate the impact of the CM intervention in 22 communities (11 intervention) in the study catchment area. To measure the impact of the intervention and monitor changes in study outcomes and the CM domains in each community, two cross-sectional, population-based surveys were implemented among approximately 1,200 randomly sampled adults, ages 18–35 years. The cross-sectional surveys were conducted at baseline in March-June 2012 (prior to intervention implementation) and again in July-September 2014, following approximately two years of intervention implementation. Serial cross-sectional samples were chosen instead of a panel design due to high levels of in- and out- migration in the study population as well as the advantages for measuring community-level indicators and community change in each village.

This study was approved by the Institutional Review Boards at the University of North Carolina-Chapel Hill and University of California-San Francisco, the Human Research Ethics Committee (Medical) at the University of the Witwatersrand in South Africa, and the Mpumalanga Department of Health and Social Development Research Committee.

### Community randomization

Communities were randomized at a 1:1 ratio, resulting in 11 intervention communities and 11 control communities. In order to achieve a balanced allocation of control and intervention communities with respect to covariates hypothesized to be associated with the primary outcome, gender norms, a restricted randomization scheme was employed [36]. First, all possible two-arm allocations were enumerated and examined to see how they balanced with respect to community characteristics from the 2011 AHDSS that might be associated with gender norms: community population size, average education, number of working residents in the community, number of foreign residents, proportion temporary migrants, and proportion female headed households. A reduced list of 50 allocations from the total 466,918 restricted allocations was randomly chosen and one was selected by a community representative during a community meeting. Community representatives and leaders from all of the communities agreed to participate in the study.

### Survey sampling

The sampling frame for the baseline and endline surveys was the 2011 and the 2013 Agincourt HDSS annual census, respectively, limited to households with 18–35 year old residents, and then stratified by gender to create ‘male’ and ‘female’ sampling frames. The target enrollment for each of the 22 communities (11 control and 11 intervention) was 55 individuals with 27–28 males and females per community. Only one individual per home was sampled for recruitment. Eligibility criteria for participation in the survey included: resides in the home (spends a majority of nights in a 7-day week within the home), is 18–35 years of age per confirmed date of birth, is the gender assigned to the home (i.e., male or female) and has lived in the study area for the past 12 months. Written informed consent was obtained from all participants in the survey and was offered in the local language, Shangaan, or English. The survey was interviewer-administered in Shangaan or English using Computer Assisted Self-Interview (CAPI) in which the interviewer directly entered responses into a tablet computer.

### Measures

Exposure is defined as the community randomization assignment. The primary endpoint for assessing intervention impact is gender norms, as measured by the Gender Equitable Men’s Scale (GEMS). This scale was originally developed in Brazil and has been adapted for use in the African context [37]. The scale used in the present study was based on an Ethiopian version with 24 items, which achieved high internal consistency reliability [38]. Wording of select items was changed in consultation with local research team members to increase appropriateness for the local context. A split-sample exploratory and confirmatory factor analysis was conducted using baseline data and resulted in unidimensional scales for men (17 items) and women (13 items), with higher scores representing more equitable gender norms. Items fell into four content areas: sexual relationships (e.g., “Men are always ready to have sex”); violence (“A woman should tolerate violence to keep her family together”); reproductive health and disease prevention (“It is a woman’s responsibility to avoid getting pregnant”); and household roles and decision-making (“A man should have the final word about decisions in his home”). Individuals’ scores were the sum of all items (each item ranged from 1–3).

Secondary endpoints for the study include having multiple (two or more) sexual partners in the last 12 months, sexual partner concurrency (two or more ongoing sexual partnerships within last three sexual partners), inconsistent condom use with last three partners, intimate partner violence (perpetration and experienced), and recent hazardous/harmful drinking. IPV victimization and perpetration was defined as reporting at least one of seven types of physical and sexual IPV on a World Health Organization questionnaire adapted for South Africa [39]. Hazardous/harmful drinking was defined as a score of 8 or above using the Alcohol Use Disorder Identification Test (AUDIT) [40]. Measures of community mobilization were also developed by the research team to monitor change processes in each of the 6 mobilization domains; the development and performance of these measures is reported elsewhere [41].

### Sample size

Power and sample size needed to detect changes in the primary endpoint of interest, the GEMS score, were determined for the male and female population. Assuming a sample of 600 subjects in each of the two arms, an intra-cluster correlation of 0.05 and an average cluster size of 55 resulted in an estimated design effect of 3.7, thus reducing the effective sample size to 162 for each arm. For men, with an effective sample size of 81 and estimated GEMS standard deviation of seven (score range of 17–51), the estimated power to detect a four-point increase in GEMS scores was 91 %. For women, with an effective sample size of 87 and an estimated standard deviation of six (score range of 13–39), the estimated power to detect a three-point increase was 80 %.

### Planned endline analysis

For the primary data analysis to determine the effect of the intervention, an intent to treat (ITT) approach will be used, with both community and individual as the unit of analysis. For analyses with individual as the unit of analysis, random effects models and/or generalized estimating equations with robust variance estimators will be used to account for clustering and ensure correct standard errors. All analyses will be weighted and conducted separately for men and women. Multivariate analyses for the primary outcome (endline GEMS score) and secondary outcomes will control for appropriate community characteristics at baseline, such as an aggregate village-level GEMS score.

## Discussion

This is one of the first randomized community trials of an intervention using community mobilization to address the intersection of HIV and inequitable gender norms. In the area of HIV prevention, community mobilizing interventions have demonstrated successes in increasing condom use, [3, 23, 29, 30, 42, 43] improving service access and quality, [3, 44] increasing social capital or social cohesion, [3, 45] and in promoting uptake of HIV counseling and testing [46]. Though a number of successful projects have spurred interest in the application of community mobilization for HIV prevention, most interventions utilizing mobilization to date have lacked a clear theory-based, conceptual framework or have failed to identify core CM components needed to mobilize communities around HIV prevention and care, [47, 48] something that will be critical to the scale-up of interventions that utilize mobilizing approaches. Drawing from the rich literature from sociology, community empowerment, community development, and community capacity we developed a theory based framework for community mobilization onto which we mapped and monitored our intervention activities. Ensuring that our intervention activities addressed all six theorized mobilization domains we believe increases the chances that our intervention will have an impact on the desired outcomes. In addition, as we have developed a validated, quantitative measure of community mobilization (comprised of six theorized CM domains), we will also be able to assess whether changed negative gender norms occurred through the theorized CM domains and which domains were most relevant to change.

This study has a number of strengths. First, a carefully designed and theory-driven intervention was used. Second, the intervention is being rigorously evaluated using a cluster randomized controlled trial. Third, the study is based in a demographic surveillance site which provided a sampling frame and key demographic information on the target population, including a longstanding delineation of village boundaries that is often difficult for studies to establish. Fourth, this was a true collaboration between an existing Non-Governmental Organization and Academic/Research partners allowing for the adaptation of an existing intervention, the OMC Campaign, to better align with the theory of CM and ensure rigorous monitoring and evaluation. Finally, intervention targets were set for intervention dose and reach and monitored using monthly and quarterly reports which helped ensure that the target population was reached with the intervention activities theorized to be most salient to changing gender norms.

The study also has limitations. First, the study area is characterized by high levels of in- and out-migration, mainly for work purposes. It is estimated that 33 % of men and 19 % of women in the study area are temporary migrants [33]. As a result, eligibility for survey participation included residency in the study community for the past 12 months to ensure sufficient intervention exposure at the endline survey (at which time the same eligibility criteria applied). While migrant men were not excluded from participating in intervention activities, as they are a key population to reach, they were not included in the surveys and thus we cannot make inferences about the acceptability or impact of the intervention among migrants. Second, as with all community randomized trials, there is the potential for contamination. Significant changes to Sonke’s OMC intervention model were made to reduce contamination, including elimination of mass media engagement, which is typically a central strategy in Sonke’s work. This aspect of the OMC adaptation may weaken the effect of the intervention. Other strategies to reduce contamination included ensuring that mobilizers were living in one of the intervention communities, and for some events such as soccer tournaments, being careful about where activities were conducted and which communities were invited. To measure the magnitude of contamination, intervention exposure was measured both in intervention and control communities during the endline survey.

## Conclusion

This is one of the first randomized controlled trials to test a theory-based model of community mobilization to change gender norms and prevent HIV. Determining whether this CM intervention is effective in changing harmful norms and reducing high levels of HIV risk behaviors particularly among men in this population is a matter of considerable importance for South Africa. The intervention is based on an adaptation of a widely used intervention (the One Man Can campaign) supported by substantial on-the-ground knowledge of an established, local NGO, infrastructure that could enable scale-up and sustainability in South Africa if the intervention is found to be effective.
